# The Functionality of Endothelial-Colony-Forming Cells from Patients with Diabetes Mellitus

**DOI:** 10.3390/cells9071731

**Published:** 2020-07-20

**Authors:** Caomhán J. Lyons, Timothy O’Brien

**Affiliations:** Regenerative Medicine Institute (REMEDI), CURAM, National University of Ireland Galway, H91 TK33 Galway, Ireland; c.lyons15@nuigalway.ie

**Keywords:** endothelial colony forming cells, cell modification, diabetes, disease-related cell dysfunction

## Abstract

Endothelial-colony-forming cells (ECFCs) are a population of progenitor cells which have demonstrated promising angiogenic potential both in vitro and in vivo. However, ECFCs from diabetic patients have been shown to be dysfunctional compared to ECFCs from healthy donors. Diabetes mellitus itself presents with many vascular co-morbidities and it has been hypothesized that ECFCs may be a potential cell therapy option to promote revascularisation in these disorders. While an allogeneic cell therapy approach would offer the potential of an ‘off the shelf’ therapeutic product, to date little research has been carried out on umbilical cord-ECFCs in diabetic models. Alternatively, autologous cell therapy using peripheral blood-ECFCs allows the development of a personalised therapeutic approach to medicine; however, autologous diabetic ECFCs are dysfunctional and need to be repaired so they can effectively treat diabetic co-morbidities. Many different groups have modified autologous diabetic ECFCs to improve their function using a variety of methods including pre-treatment with different factors or with genetic modification. While the in vitro and in vivo data from the literature is promising, no ECFC therapy has proceeded to clinical trials to date, indicating that more research is needed for a potential ECFC therapy in the future to treat diabetic complications.

## 1. Introduction

### 1.1. Endothelial Colony Forming Cells

The concept of vascular regeneration using cell therapy began in 1997 with the discovery of endothelial progenitor cells (EPCs) by Asahara et al. [1]. These EPCs were shown to be spindle shaped and express CD34^+^, CD45^+^, CD31^+^, vascular endothelial growth factor receptor 2^+^ (VEGFR2^+^), and Tie-2^+^. Asahara’s paper showed that these EPCs demonstrated endothelial characteristics such as being able to uptake acetylated-low density lipoprotein, and their data also suggested that EPCs could integrate with the host vasculature once transplanted. In the following years, a vast body of research was carried out investigating the characteristics of EPCs and their potential as a vascular cell therapy, with a number of clinical trials carried out using EPCs for conditions such as liver cirrhosis, peripheral artery disease and pulmonary hypertension [2,3,4,5]. However, Asahara’s EPCs were later shown to be have a low proliferative capacity, to be of myeloid origin rather than endothelial origin, with high expression of the immune markers CD14 and CD45, and to promote angiogenesis by paracrine mechanisms rather than direct integration or tube formation [6,7]. Instead, Ingram et al. [8] identified a novel cell type termed endothelial colony forming cells (ECFCs) which has recently been suggested as a cell type more akin to the ‘endothelial progenitor cell’ than Asahara’s EPCs in a consensus paper by Medina et al. [9].

**Figure 1:**
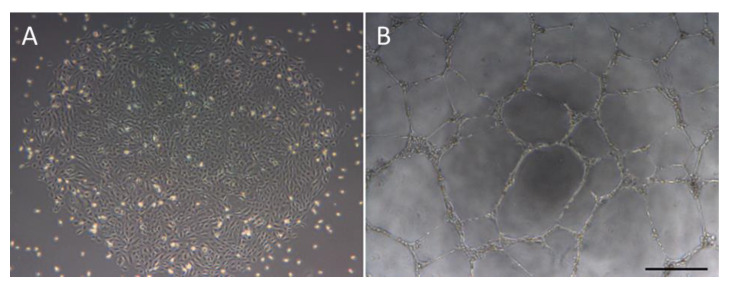
Morphology of endothelial-colony-forming cells (ECFCs) and their ability to form tubes. A = cobblestone morphology of ECFC colonies. B = In vitro tubulogenesis assay showing the ability of ECFCs to form a network of tubes. Scale bar = 500 µm.

ECFCs, also referred to as blood outgrowth endothelial cells, late outgrowth endothelial cells, or late EPCs, are a rare progenitor cell population which can be isolated from both peripheral blood (PB) and umbilical cord (UC), with a predicted frequency of 1.7 ECFCs per 1 × 10^8^ peripheral blood mononuclear cells (PBMCs) [10]. When initially plated, ECFCs are cultured on collagen type I coated flasks and colonies with the distinctive endothelial-like cobblestone morphology typically appear in culture after day 6 for UC-derived ECFCs, or day 15 for PB-derived ECFCs [8,11] (Figure 1). ECFCs were found to express the endothelial markers CD31, CD146, VEGFR2, and von Willebrands factor, and to be negative for the immune cell markers CD45 and CD14, while also being positive for the stem cell marker CD34 [12].

Functionally ECFCs were shown to have a high proliferative capacity and to possess the ability to form new vascular tubes in vitro [8,13]. When ECFCs were tested in in vivo plug models they also produced functional vessels in vivo and possessed the ability to integrate with pre-existing host vasculature [10,14]. However, with much confusion in the field between Asaharas’ EPCs and Ingram’s ECFCs a consensus paper was published by Medina et al. [9] in an effort to standardize ECFCs and to avoid any confusion with Asahara’s EPCs. This consensus paper defined ECFCs as being CD31^+^, CD34^+^, VEGFR2^+^, and CD45^-^ while also possessing the ability to produce vasculature, both in vitro and in vivo, and retaining a high proliferative capacity (Table 1). The ability of ECFCs to both form vessels and integrate into pre-existing vasculature, while having a high proliferative capacity makes them an optimal candidate for a potential cell therapy for vascular disorders.

With ECFCs having demonstrated their functional capacity in vitro and in in vivo plug assays, numerous pre-clinical studies were then carried out using ECFCs to treat a plethora of pathologies, in particular ischaemic disorders such as ischaemic brain [15], ischaemic retinopathy [16], and hind-limb ischaemia (HLI) [17,18]. Once ECFCs are injected into mice both Ding et al., [19] and Reid et al. [16] showed that they can home to the site of injury in a model of stroke and a model of retinopathy, respectively. Additionally, ECFCs demonstrate efficacy in improving ischaemic conditions in models. Examples of this include a study by Kang et al. [17], who transplanted UC-ECFCs into the ischaemic muscle a mouse model of HLI and demonstrated improved blood flow. Similar improvement was observed by Burger et al. [20], who demonstrated that transplanted UC-ECFCs can improve ischaemic kidney disease in mouse models via an anti-apoptotic and brush border protective effect on the renal tubule cells. In addition, the study by Ding et al. [19] showed that inter-arterial injection of UC-ECFCs into mouse models of stroke can improve angiogenesis at the site of injury. This resulted in improved stroke recovery, increased neurogenesis and reduced apoptosis in mouse models. In a murine model of traumatic brain injury Huang et al. [21] demonstrate that intracerebroventricular transplantation of UC-ECFCs gives rise to an increased microvessel density and less pathological extravasation in the blood brain barrier compared to saline controls which resulted in improved modified neurological severity score. Reid et al. [16] transplanted UC-ECFCs intravitreally at varying doses into an oxygen induced retinopathy mouse model which led to a significantly higher vascularization compared to saline control. Furthermore, when transplanted via the carotid artery they also showed homing to the site of injury and improved vascularization in the ipsilateral retina. Similar recovery was observed by the same group in the same model using PB-ECFCs [13]. The above improvements in preclinical murine models have been attributed to neoangiogenesis, direct integration of ECFCs into pre-existing vasculature, and the release of exosomes which promote nutrient and oxygen delivery, thereby facilitating healing and functional recovery of the ischaemic area [10,20]. The data presented above demonstrates that ECFCs can facilitate functional recovery in a variety of preclinical ischaemic disease models however to date no clinical trials have been carried out using either PB-ECFCs or UC-ECFCs.

For a future cell therapy, the source of ECFCs becomes an important question. UC-ECFCs are noted to hold many advantages over PB-ECFCs, including the appearance of colonies sooner in culture, the generation of more colonies, and a higher proliferation capacity [8,22]. This indicates that an allogeneic therapy using UC-ECFCs may be the more attractive therapeutic option in the future; however, PB-ECFCs can be autologously derived, and this offers distinct advantages as it facilitates the development of a personalized cell therapy and also negates the need for immunosuppressive agents which are required for many allogeneic cell therapy approaches to prevent an alloimmune response.

### 1.2. Diabetes Mellitus

Diabetes mellitus (DM) is a metabolic condition where there is a lack of insulin production (type 1) or a combination of impaired insulin secretion and action (type 2). Currently, there is an estimated 463 million people living with DM worldwide, and it is projected that 578.4 million will be affected in 2030 [23], indicating that DM is a global health concern which is exponentially growing. Both type 1 and type 2 DM lead to chronic hyperglycaemia in patients which is due, at least in part, to impaired cellular uptake of glucose. Low glucose uptake has also been shown in endothelial cells from diabetic patients with a resultant impairment in functionality being noted [24]. The resulting dysfunctional endothelial layer plays a major role in atherogenesis by facilitating the build-up of an atherosclerotic plaque on the inner lining of the blood vessels, which gives rise to a plethora of ischaemic conditions affecting the heart, brain and lower extremity (macrovascular disease). Atherosclerosis involving the lower extremities leads to peripheral artery disease [25], this can progress even further and patients can develop critical limb ischaemia whereby the patient develops rest pain and ischaemic non-healing ulcers which often lead to amputation of the affected limb [26]. DM is also complicated by the so-called microvascular complications of retinopathy, diabetic kidney disease, and peripheral neuropathy [25,27]. With the vasculature being a necessary highway for nutrient and oxygen delivery, DM is described as a system-wide dysfunction that leads to the diverse array of complications described above. Therefore, with a pathological vascular system playing a major role in the pathogenesis of these co-morbidities, ECFCs are a prime cell therapy candidate to treat these vascular complications.

## 2. Diabetic ECFCs

While ECFCs have been investigated for a number of different conditions, little work has been carried out on the potential for ECFCs to resolve diabetic complications, and with few treatment options available for those affected by severe DM complications, e.g., critical limb ischaemia; this makes ECFCs a strong cell therapy candidate. Ideally, an autologous cell therapy with PB-ECFCs as a cell source would be the favourable cell therapy approach, removing the need for immunosuppressant treatment; however, recent studies have shown that ECFCs from patients suffering from DM can have a dysfunctional phenotype [28,29]. This indicates that DM ECFCs may have a reduced therapeutic effect compared to ECFCs derived from healthy patients. There is also concern that cell isolation and propagation to required numbers for a cell therapy may be problematic in patients with DM although this has not been explored extensively in the literature.

### 2.1. ECFCs from Diabetic Patients

A number of different studies have shown that ECFCs from patients suffering from DM have an altered functionality compared to ECFCs derived from healthy donors. Jarajapu et al. [30] reported a reduced isolation rate for DM ECFCs (30%) compared to ECFCs from healthy controls (90%) however more data are required to confirm that DM ECFCs have a reduced frequency than healthy controls. Functionally DM ECFCs were noted to have reduced tubulogenic capacity, reduced migration toward SDF-1 and VEGF-B (two of the main ECFC chemotactic factors), and reduced proliferation in vitro [28,31]. Ho et al. [32] demonstrated that DM PB-ECFCs, when co-cultured with human umbilical vein endothelial cells (HUVECs), resulted in a reduced number of tubules in vitro compared to healthy controls. While DM PB-ECFCs have yet to be analysed in an in vivo plug model, Leicht et al. [29] injected both DM PB-ECFCs and healthy age matched control PB-ECFCs intraveneously into a mouse model of HLI. Healthy control ECFCs resulted in a significantly higher blood limb perfusion, in contrast DM PB-ECFCs only resulted in a modest increase in the blood perfusion, which was not significantly different from the untreated control indicating a dysfunctional phenotype in DM ECFCs compared to ECFCs from healthy controls. All the above confirm the reduced functionality of ECFCs from DM patients in vitro and the reduced functionality in vivo. It also highlights the importance of understanding the pathophysiology of diabetes induced ECFC dysfunction to facilitate the use of these cells as an autologous cell therapy in the future.

### 2.2. UC-ECFCs from Gestational Diabetes Pregnancies

A number of studies were carried out on UC-ECFCs from mothers with gestational DM (G-DM) due to the ease of isolation of ECFCs from UC tissue and availability of the tissue source. These studies were carried out to provide some insight into the dysfunction associated with ECFCs exposed to a diabetic environment. The research on UC-ECFCs from G-DM pregnancies has shown that the diabetic intrauterine environment also reduces the number of UC-ECFC colonies and their functionality in the form of reduced tubulogenic capacity, reduced proliferation, reduced migration and that G-DM UC-ECFCs have reduced sirtuin1 and sirtuin3, which are involved with regulating cell metabolism and aging, compared to UC-ECFCs from healthy pregnancies [33,34]. Dincer [35] demonstrated that G-DM UC-ECFCs were less tolerant to hypoxic environments, resulting in increased senescence and decreased tubulogenesis in hypoxic conditions compared to ECFCs from healthy controls. Acosta et al. [36] showed no difference in the frequency of UC-ECFC between G-DM and healthy pregnancies but carried out no functional analysis on the isolated UC-ECFCs. While there is variability in the results of UC-ECFCs from G-DM pregnancies, most confirm the decreased functionality shown above in DM ECFCs from peripheral blood.

### 2.3. Effect of High Glucose Conditions on ECFCs In Vitro

The above data using DM and G-DM ECFCs correlates with in vitro models of ECFCs which were cultured in high glucose conditions. When UC-ECFCs are cultured in high glucose conditions they have reduced tubulogenesis and migration, and these effects increased with an increasing hyperglycaemic environment [33,37]. These studies support the hypothesis that DM related hyperglycaemia is one of the main causes of ECFC dysfunction.

In addition, while this review focuses on the effect of DM on the functionality of ECFCs, dysfunctional ECFCs can also be observed from a number of other pathological conditions such as chronic obstructive pulmonary disorder, venous thromboembolic disease, and moyamoya disease [38,39,40].

The results presented above suggest that ECFCs derived from a diabetic environment are a poorly suited cell source for an autologous therapy due to their dysfunctional phenotype. Therefore, for the development of an autologous therapy using ECFCs from DM patients, the pathophysiology of DM ECFCs must be analysed to identify a potential target to improve DM ECFC functionality and maximize their therapeutic potential.

### 2.4. Pathophysiology of ECFC Dysfunction in Diabetes Mellitus

**Figure 2:**
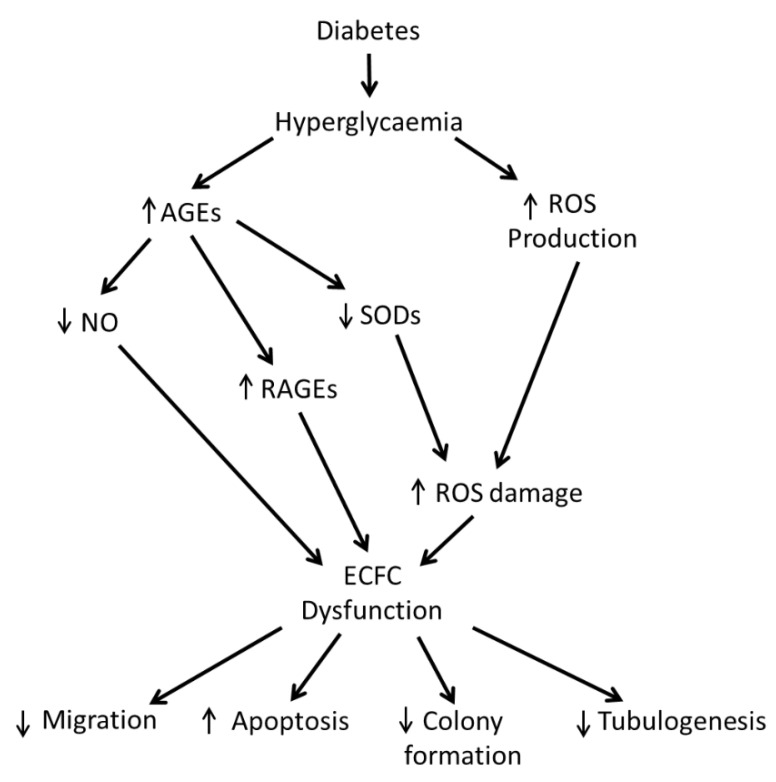
Mechanisms promoting diabetes mellitus (DM) related ECFC dysfunction. AGEs = advanced glycation end-products, NO = nitric oxide, RAGEs = receptor for advanced glycation end-products, ROS = reactive oxygen species.

In DM, the associated hyperglycaemia facilitates the glycation of lipids and proteins in the blood, resulting in the formation of advanced glycation end-products (AGEs) such as AGE-albumin [41] (Figure 2). AGEs are known to damage the vascular endothelium and they have recently been shown to negatively affect ECFCs. ECFCs exposed to AGEs were found to have reduced functionality in the form of reduced migration, tubulogenic capacity and adhesion [42]. In addition, AGEs have been shown to cause ECFCs to upregulate the AGE receptor and to downregulate proteins involved in vascular homeostasis such as protein kinase B, which is involved with NO production, and superoxide dismutase (SOD), which functions to reduce convert the reactive oxygen species (ROS) superoxide into molecular oxygen and hydrogen peroxide which is then subsequently broken down to water [41,42,43]. Endothelial cells themselves are noted to naturally produce ROS, which is an important signalling mediator for a number of angiogenic processes such as migration [44]. However, high levels of ROS have been shown to damage endothelial cells [45,46]. Excessive levels of ROS are produced as a result of the DM related hyperglycaemia. High levels of ROS result in an inflammatory environment in vessels which damages the endothelium and with SOD downregulated by AGEs ECFCs are less capable of reducing ROS levels. Furthermore, superoxide reduces the bioavailability of NO by converting it to ONOO^-^ which itself causes vascular aging [47,48]. Much of the reduced cell functionality noted from increased levels of AGEs and excessive levels of ROS match the reduced functionality observed in DM ECFCs, therefore suggesting that AGEs and excessive levels of ROS are some of the leading causes of ECFC dysfunction in DM. Consequently, targeting some of these pathways may improve DM ECFC function to improve their therapeutic efficacy.

While much work has been carried out on the pathophysiology of DM-related defects in ECFCs, there is still a lot unknown about the mechanisms behind these defects. More work is needed to identify a potential molecular target to upregulate/downregulate to improve the functioning of DM ECFCs in order to facilitate their use as an autologous therapy for diabetic complications.

### 2.5. Repair of ECFC Diabetic Related Dysfunctionality

As demonstrated above ECFCs have been shown to be deficient in DM [28], which inhibits their use as an autologous therapy; therefore, to bypass this issue, a number of research groups have investigated the potential of modifying ECFCs to improve their function. Cells can be modified using a variety of techniques such as pre-treating the cells with certain factor(s) to improve the cells function or with genetic modification to upregulate/downregulate certain proteins or pathways.

An example of DM ECFC modification is by Ho et al. [32], who used conditioned media from human embryonic stem cell derived-endothelial cells (ESC-ECs) to improve the tubulogenic function of DM ECFCs. They co-cultured DM ECFCs with HUVECs for an in vitro tubulogenesis assay and while an improvement in the tubulogenic capacity of DM ECFCs was observed using the ESC-EC conditioned medium, it is difficult to determine whether the improved tubulogenesis was via direct tube formation with ECFCs or via the paracrine effects of ECFCs on HUVECs. Regardless, this study showed functional improvement in DM ECFCs; however, additional functionality assays such as growth kinetics and in vivo angiogenic capacity need to be examined on these modified DM ECFCs to further investigate their therapeutic potential. Furthermore, the ethical issues associated with ESC-ECs may limit the therapeutic potential of this modification technique.

Gui et al. [33] showed that the dysfunctional phenotype of UC-ECFCs from G-DM pregnancies can be repaired with 10 nM 1.25 (OH)_2_ vitamin D_3_ so that the G-DM UC-ECFCs showed similar 2D tubulogenesis and migration capabilities as UC-ECFCs from healthy pregnancies. 10 nM 1.25 (OH)_2_ vitamin D_3_ also improved the migration and in vitro tubulogenic capacity of healthy UC-ECFCs. As this modification uses a vitamin which is already commercially available it has strong therapeutic potential. However, this study only had N = 1 in the control group, and therefore, more research is needed in the form of repeating previous in vitro results with a higher control sample number, to confirm the above results, and the inclusion of an in vivo experiment to confirm functionality in an animal model before moving forward with this potential therapy.

In their paper, Wang et al., [28] showed that the reduction in tubulogenic capacity and migration capability associated with DM PB-ECFCs can be repaired using far infrared treatment and that the degree of repair correlates with the duration of far infrared treatment. This repair was in the form of increased tubulogenesis and migration compared to untreated DM PB-ECFCs. This repair was caused by reducing the levels of miRNA-134 which was upregulated in DM PB-ECFCs and miRNA-134 is noted to impair angiogenic function in ECFCs. In addition, increased cell proliferation was noted for a number of days after far infrared treatment, but this effect was lost with time. Far infrared therapy has a distinct advantage as a modification technique as it is already commercially used to improve chronic wound healing and peripheral circulation [49], but more research is needed to examine the effect of far infrared treatment on ECFCs and the angiogenic potential of these modified cells in vivo before progressing to a clinical trial.

A paper by Langford-Smith et al. [50] demonstrated an improvement in the migration capacity of DM PB-ECFCs from patients also presenting with neuroischaemic or neuropathic ulcers through the use of a novel glycomimetic C3; however, only DM PB-ECFCs from patients with neuroischaemic ulcers showed increased tubulogenesis with glycomimetic treatment. It is important to note that this study did not analyse the effect of their glycomimetic C3 on the proliferation, senescence, or the in vivo angiogenic potential of these modified DM PB-ECFCs, which would be important considerations prior to a therapeutic use of their novel glycomimetic.

Leicht et al. [29] showed that pre-treatment of DM PB-ECFCs with adiponectin resulted in an improved proliferation and migration capacity. In addition, they showed a reduction in the oxidative stress in adiponectin treated DM PB-ECFCs compared with untreated DM PB-ECFCs. While this group did not analyse the in vitro tubulogenic capacity of their modified DM PB-ECFCs, they instead analysed the repair capacity of the modified DM PB-ECFCs in an HLI mouse model. The results of this showed that untreated DM ECFCs had reduced blood flow recovery compared to healthy control ECFCs; and that there was no significant difference in the blood flow recovery reported between the adiponectin treated DM PB-ECFCs and healthy control PB-ECFCs. This shows that adiponectin treatment can restore the function of DM PB-ECFCs both in vitro and in vivo.

Based on the aforementioned studies the modification method with the most therapeutic potential is the 1.25 (OH)_2_ vitamin D_3_ treatment. While both the C3 glycomimetic by Langford-Smith et al. [50] and the adiponectin pre-treatment by Leicht et al. [29] show promising data 1.25 (OH)_2_ vitamin D_3_ is already commercially available and well-studied, but 1.25 (OH)_2_ vitamin D_3_ will need to be tested on DM PB-ECFCs to confirm the effects observed in G-DM UC-ECFCs, and a minimum of three controls would be needed to confirm the presence of a significant difference between the groups. The beneficial effects of the far infrared treatment of ECFCs appear to be transient in nature and the application of far infrared therapy topically to continue the beneficial effect on ECFCs in vivo has not yet been tested, and while the addition of conditioned media from ESC-ECs improved the function of DM PB-ECFCs it may encounter ethical issues in its translation to the clinic. While much research has been carried out on autologous DM ECFC modification, no treatment has progressed into a clinical trial yet, indicating that new approaches are needed to tackle DM PB-ECFC dysfunction to facilitate their use clinically.

## 3. Allogeneic ECFC Therapy for Diabetic Complications

An alternative cell therapy approach would be to use an allogeneic cell source, such as UC-ECFCs, to treat diabetic complications. While there has been positive data using UC-ECFCs in a number of ischaemic disease models such as brain ischaemia and HLI [17,18,19], little research has been carried out on UC-ECFCs in diabetic models. Siddiquee et al. [51] conducted a study whereby UC-ECFCs were injected into the corpus cavernosum of a rat model of diabetic erectile dysfunction and they demonstrated improved intra-carvernosum pressure, increased endothelial nitric oxide synthase, improved cavernosal nerve function and reduced cavernosum apoptosis, all of which resulted in functional recovery. This study suggests that UC-ECFCs can promote functional recovery in diabetic models.

In contrast to the un-modified UC-ECFCs used by Siddiquee et al. [51], Mena et al. [37] used a combined modification-allogenic approach in a diabetic model. Mena et al. [37] preconditioned UC-ECFCs in acidic conditions and demonstrated that these preconditioned UC-ECFCs showed improved adhesion and increased resistance to endogenous cytotoxic compounds in vitro, such as tumour necrosis factor α and monosodium urate crystals. The preconditioned UC-ECFCs were also more resistant to high glucose related cell dysfunction compared to non-preconditioned UC-ECFCs. When both preconditioned and non-preconditioned UC-ECFCs were transplanted into a diabetic mouse model of HLI both significantly improved the vascularisation and reduced the inflammation, but the preconditioned UC-ECFCs had significantly higher recovery than non-preconditioned ECFCs.

Another allogeneic approach would be the generation of induced pluripotent stem cell (iPSC) derived cells. Prasain et al. [52] developed a novel protocol for the generation of iPSC-ECFCs which possessed similar morphology in vitro and similar angiogenic capacity in vitro and in vivo as UC-ECFCs. They reported a protocol which could convert a single iPSC into >10^8^ iPSC-ECFCs, demonstrating the expansion capability of iPSC technology for ECFCs, which typically suffers from a low frequency in PB. This high expansion rate could facilitate the banking of an allogeneic iPSC-ECFC therapy or the generation of a personalized autologous cell therapy approach, however iPSC-ECFCs will need to first be tested in a diabetic model to determine whether they can resist the severe diabetic environment and promote recovery. In addition to iPSC-ECFCs, iPSC-endothelial cells have been extensively explored in the literature as a therapeutic option for vascular disorders and has shown positive results in a variety of pre-clinical models including oxygen induced retinopathy, HLI and wound healing [53,54,55,56]. Despite the promising results of iPSC derived cells, it is important to consider the long culture times association with the generation of iPSC derived cells and the ability of iPSCs to form a teratoma upon transplantation which may hinder the progression of this therapeutic option. However, a paper by Margariti et al. [57] developed a novel protocol which shortened the time it takes to make iPSC-endothelial cells with these cells showing no ability to form a teratoma upon transplantation, while also demonstrating increased blood flow recovery in a HLI model compared to mice who received fibroblasts alone or medium alone. To date, this iPSC development technique has yet to be applied to the generation of iPSC-ECFCs to decrease the time necessary to create a potential ECFC therapy.

As previously mentioned, an allogeneic treatment with UC-ECFC would require the use of immunosuppressive agents to prevent an alloimmune response. To tackle this issue a number of groups have combined ECFCs with mesenchymal stromal cells (MSCs) which has been shown to reduce the alloimmune response of allogeneic ECFCs [58] while also showing functional efficacy in HLI models [17,18,26]. In addition, MSCs have been demonstrated to both promote ECFC survival at the site of injury for up to 14 days in models of HLI [17], and improve vessel stability as MSCs are thought to form smooth muscle like cells which will stabilise the vessels formed [59]. This interaction between ECFCs and MSCs is thought to be endoglin-integrin mediated but more research is needed to confirm this [26]. Despite the promising results in non-diabetic models, the use of MSCs in combination with ECFCs has not been carried out in diabetic models to date.

Another point of consideration is that in vitro data has shown that the high glucose environment which can be found in patients suffering from DM can negatively affect normal ECFC function in as little as three days [28], as shown above. Therefore, it is possible that the healthy ECFCs may adopt the DM ECFC dysfunction phenotype upon transplantation, leading to sub-optimal recovery, which may partly explain the lack of literature on UC-ECFCs in diabetic models. In the authors’ opinion, ECFCs which are resistant to the DM environment are needed to rescue DM complications, therefore indicating that cell modification may be required for both autologous and allogeneic ECFC therapy to treat DM complications.

## 4. Conclusions

ECFCs are an attractive cell therapy for vascular regeneration which has shown efficacy in vitro and in vivo in ischaemic models. ECFCs can be derived from both UC and PB but little research has been carried out on allogeneic UC-ECFCs in diabetic models to date and the use of autologous PB-ECFCs is limited by the need to repair the functionality of DM ECFCs prior to use as a potential therapy in the future. So far, many different techniques have been used to repair DM ECFC functionality or to improve UC-ECFC function for allogeneic therapeutic use but yet there has been no clinical trial using either PB-ECFCs or UC-ECFCs. This suggests that novel research strategies are needed to develop a future ECFC based cell therapy to treat DM complications.

## Figures and Tables

**Table 1:** Difference in the characteristics between endothelial progenitor cells (EPCs) and ECFCs.

	CD31	VEGFR2	CD45	CD34	AC133	Tie-2	Tubulogenesis	Phagocytosis	Cell Morphology	Flask Coating	Appearance	Proliferative Capacity
EPCs	++	+	+	+	+	++	No	Yes	Spindle	Fibronectin	Day 4–6	Low
ECFCs	+++	+	-	+	-	+++	Yes	No	Cobblestone	Collagen I	UC = Day ~6 PB = Day ~15	UC = Very High PB = High

+ = low expression, ++ = moderate expression, +++ = strong expression, - = no expression. EPCs = endothelial progenitor cells, ECFCs = endothelial-colony-forming cells, PB = peripheral blood, UC = umbilical cord, VEGFR2 = vascular endothelial growth factor receptor 2.

## Abbreviations

AGEs	Advanced Glycation End-Products
DM	Diabetes Mellitus
ECFCs	Endothelial Colony Forming Cells
EPCs	Endothelial Progenitor Cells
ESC-ECs	Embryonic Stem Cell derived-Endothelial Cells
G-DM	Gestational Diabetes Mellitus
HLI	Hind-Limb Ischaemia
HUVECs	Human Umbilical Vein Endothelial Cells
iPSC	Induced Pluripotent Stem Cell
MSCs	Mesenchymal Stromal Cells
NO	Nitric Oxide
PB	Peripheral Blood
PBMCs	Peripheral Blood Mononuclear Cells
RAGEs	Receptor for Advanced Glycation End Products
ROS	Reactive Oxygen Species
SOD	Superoxide Dismutase
UC	Umbilical Cord
VEGFR2	Vascular Endothelial Growth Factor Receptor 2
